# Supplementary material for: Critical role of hydrogen sorption kinetics in electrocatalytic CO2 reduction revealed by on-chip in situ transport investigations
Source: Nat Commun. 2022 Nov 14;13:6911. doi: 10.1038/s41467-022-34685-9 (PMC9663515; doi:10.1038/s41467-022-34685-9)
Supplement: Supplementary file 1 — Supplementary Info [file 41467_2022_34685_MOESM1_ESM.pdf]

Supplementary Information

Critical role of hydrogen sorption kinetics in electrocatalytic CO_2 reduction revealed by on-chip in situ transport investigations

Zhangyan Mu,[1†] Na Han,[2†] Dan Xu,[3] Bailin Tian,[1] Fangyuan Wang,[1] Yiqi Wang,[1] Yamei Sun,[1] Cheng Liu,[1] Panke Zhang,[4] Xuejun Wu,[3] Yanguang Li,[2*] Mengning Ding[1*]

[1] Key Laboratory of Mesoscopic Chemistry, School of Chemistry and Chemical Engineering, Nanjing University, Nanjing 210023, China

[2] Institute of Functional Nano & Soft Materials (FUNSOM), Jiangsu Key Laboratory for Carbon-Based Functional Materials and Devices, Soochow University, Suzhou 215123, China

[3] State Key Laboratory of Coordination Chemistry, School of Chemistry and Chemical Engineering, Nanjing University, Nanjing 210023, China

[4] State Key Laboratory of Analytical Chemistry for Life Science, School of Chemistry and Chemical Engineering, Nanjing University, Nanjing 210023, China

[†]These authors contributed equally: Zhangyan Mu, Na Han.

[*]Corresponding Authors: Mengning Ding (mding@nju.edu.cn), Yanguang Li (yanguang@suda.edu.cn).

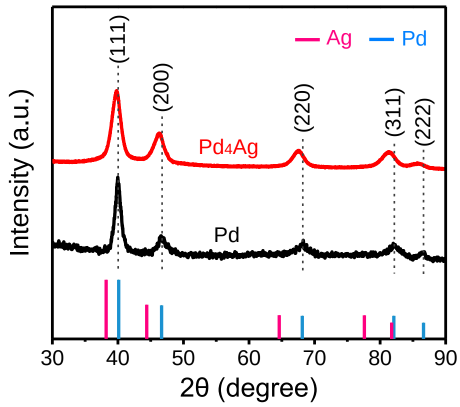

Figure S1. XRD patterns of Pd and Pd_4Ag.

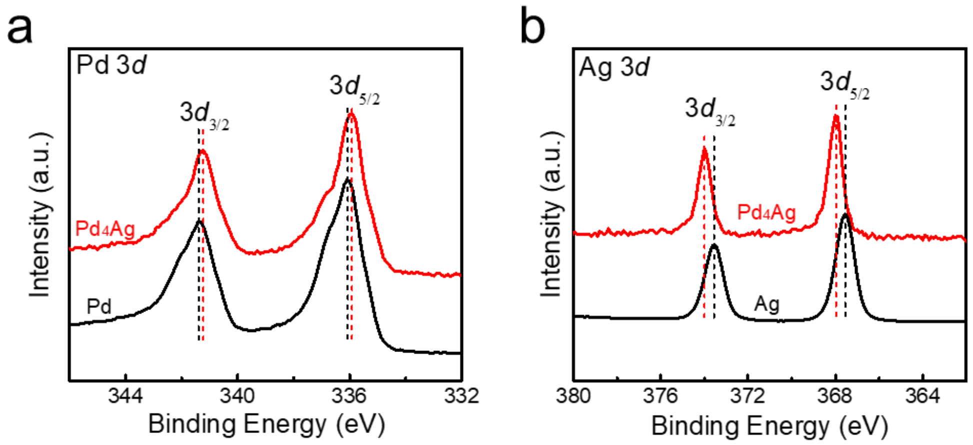

Figure S2. XPS patterns of Pd and Pd_4Ag.

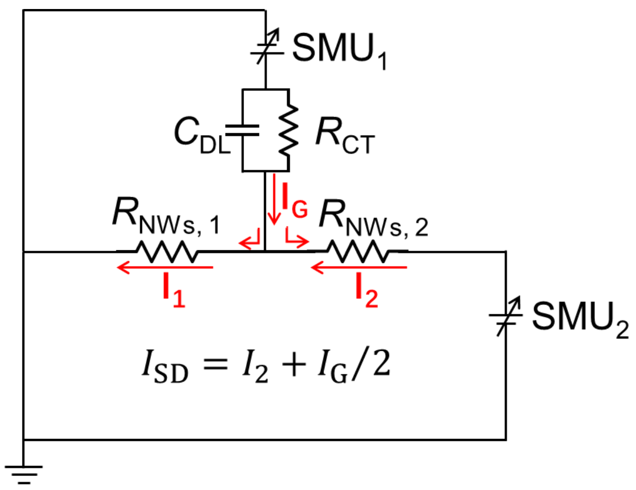

Figure S3. Equivalent circuit model of the ETS measurement. While the potential is scanned to HER and CO_2RR region, the CV current approaches several microamperes. The contributions of I_G to I_{SD} during the measurement of on-chip nanowires can be properly deducted by the equation ($I_{SD} = I_2 + I_G/2$).

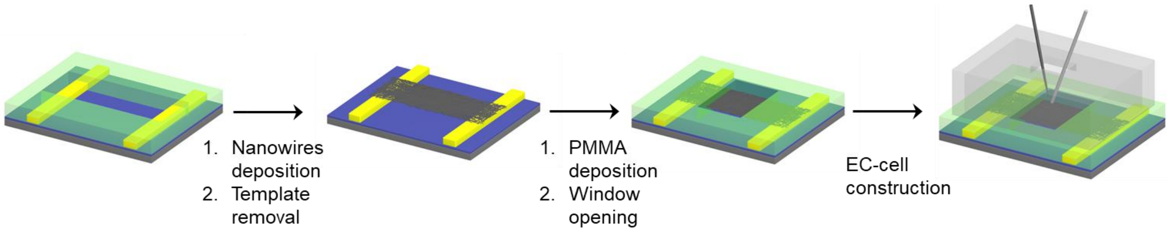

Figure S4. Schematic illustration of device fabrication and integration for the on-chip CV and ETS measurements.

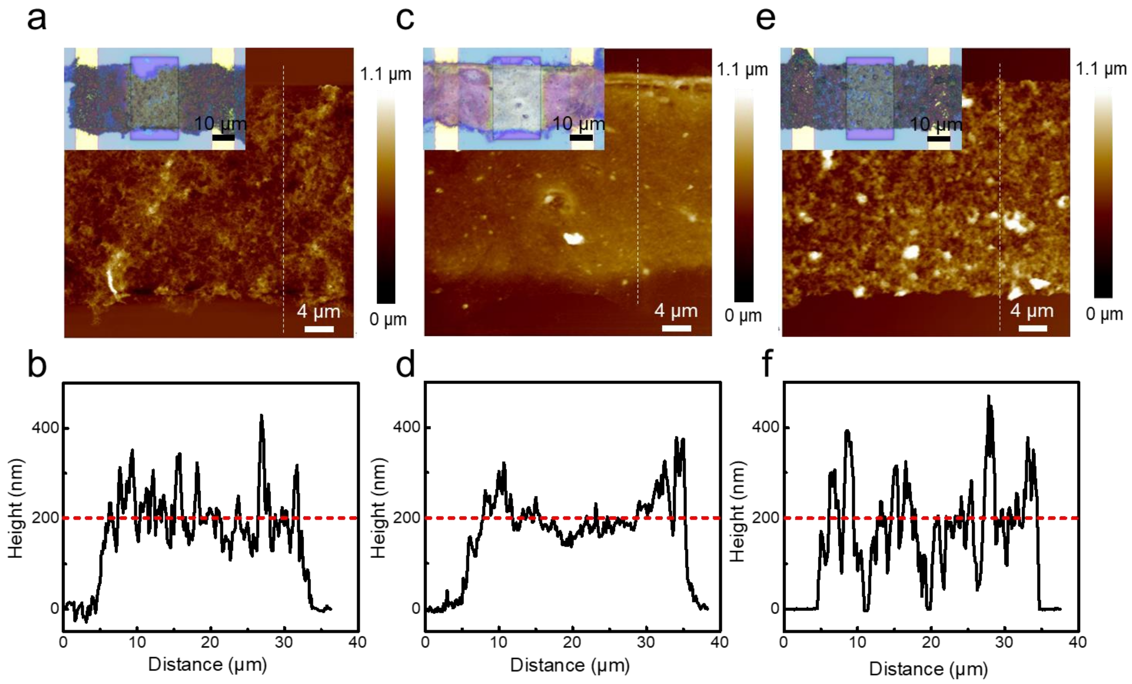

Figure S5. AFM images and section height profiles of the on-chip films of Pt (a, b), Pd (c, d) and Pd_4Ag (e, f). Insets in (a, c, e) show the optical microscopic images of the on-chip cells showing PMMA covered gold electrodes and exposed catalysts in the electrochemical windows.

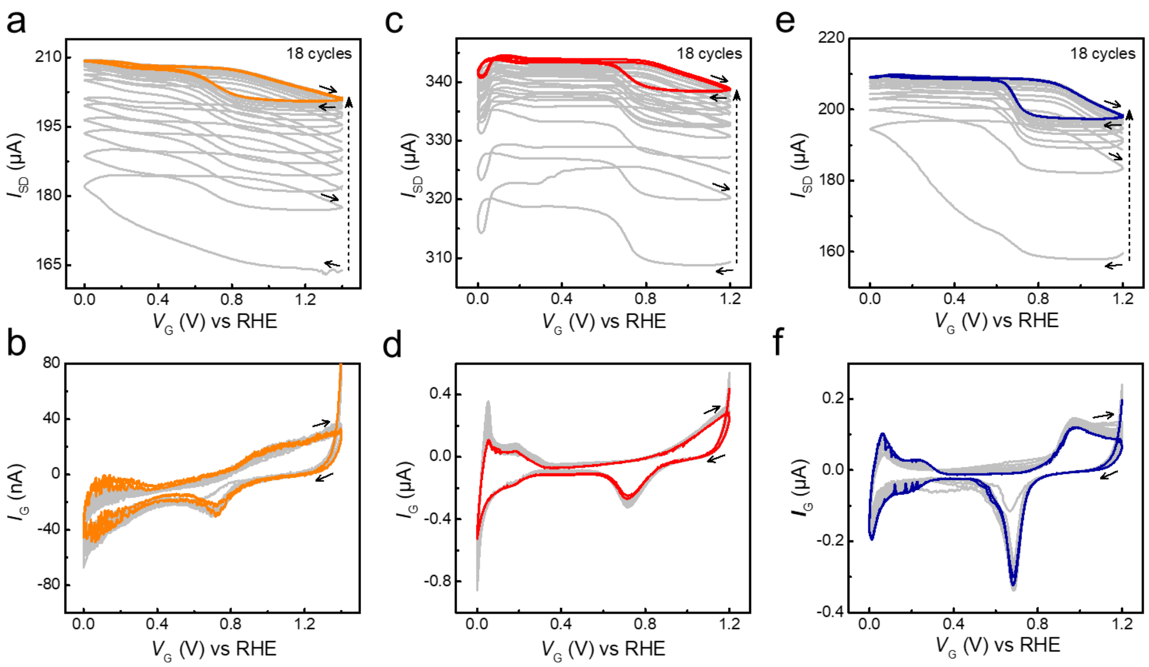

Figure S6. I_{SD}–V_G (ETS) and I_G–V_G (CV) curves during electrochemical activation process (each sweep contains two potential cycles) of Pt (a, b), Pd (c, d) and Pd_4Ag (e, f) in 0.1 M $HClO_4$ before standard CV and ETS measurements. Solid arrows in all figures indicate the potential sweeping direction.

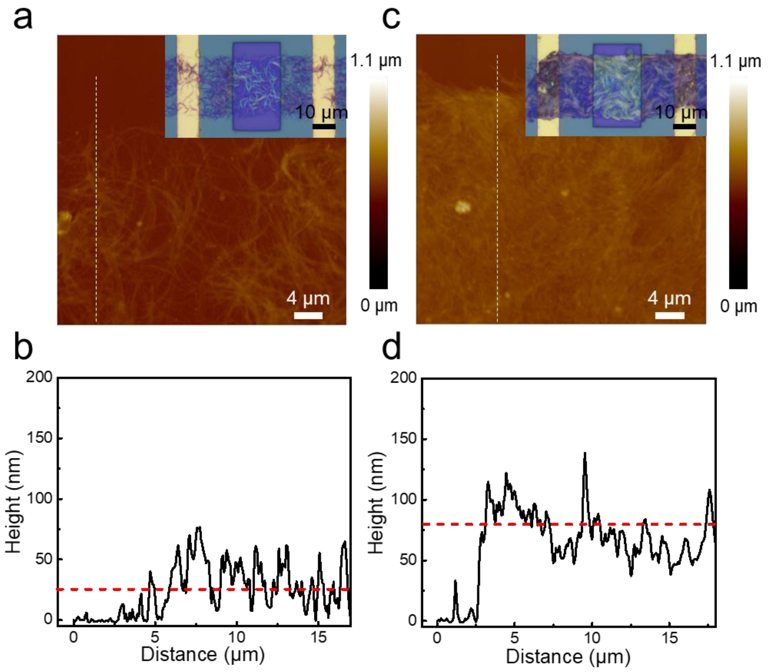

Figure S7. (a, c) AFM images of two Pd devices with thin nanowire films. Insets in (a, c) show the optical microscopic (OM) images of devices. (b) and (d) show the AFM section height profiles of the films in (a) and (c), respectively.

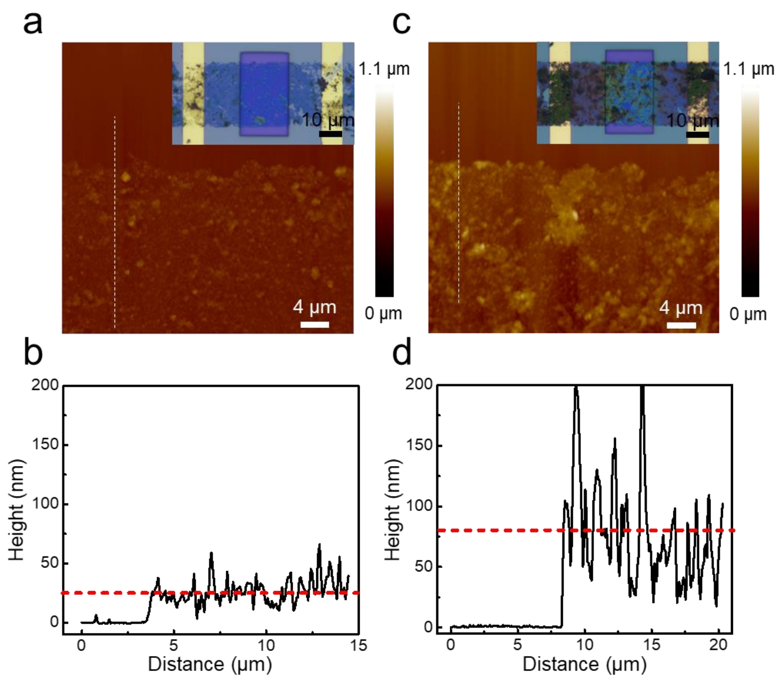

Figure S8. (a, c) AFM images of two Pd_4Ag devices with thin nanwire films. Insets in (a, c) show the optical microscopic (OM) images of devices. (b) and (d) show the AFM section height profiles of the films in (a) and (c), respectively.

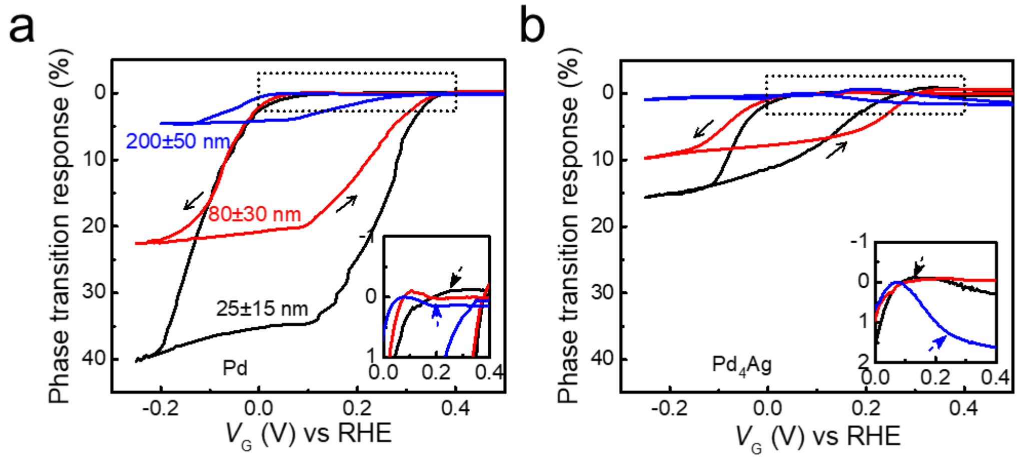

Figure S9. Phase transition responses of Pd (a) and Pd_4Ag (b) devices with different film thicknesses. Insets in (a) and (b) show the enlarged negative-potential-sweeping spectra.

Due to the insufficient electrolyte and hydrogen diffusion to the underlying layer of nanowires when the film thickness is large in device, the phase transition responses of both Pd and Pd_4Ag increase with the decreasing thickness of nanowire films. Besides, on device with thin nanowire films, hydrogen adsorption phenomenon reflected on ETS is relatively more obvious. For instance, the I_{SD} increase caused by H_{ads} is observed on Pd device with thickness of about 200±50 nm (blue dashed arrow in Figure S9a), which is however not observed with the thickness decreased to 25±15 nm (black dashed arrow in Figure S9a). Besides, the onset potential for phase transition on device with 25±15 nm film thickness is at 0.25 V_{RHE}, which is positive to the value 0.07 V_{RHE} obtained on device with film thickness of 200±50 nm. This phenomenon highly corresponds to the previous research by Duncan et al.: H adsorption and absorption both occur at 0.05~0.30 V_{RHE}, but H absorption phenomenon is relatively not significant, forming hydrogen-poor α-PdH_x[1].

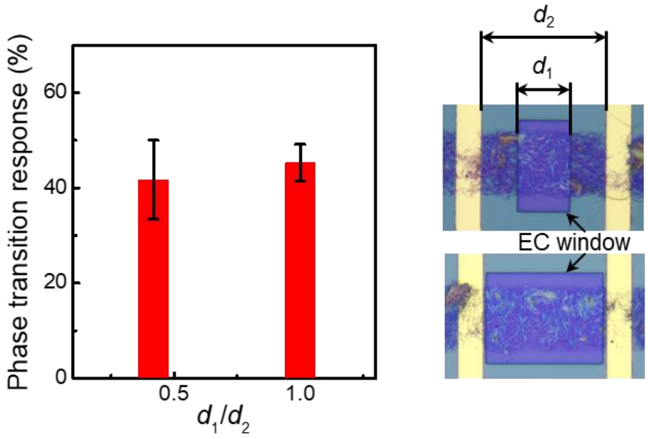

Figure S10. Phase transition responses of Pd devices with electrochemical windows of different sizes. The optical microscopic (OM) images of devices are shown on the right. The error bars present the standard error in experiments.

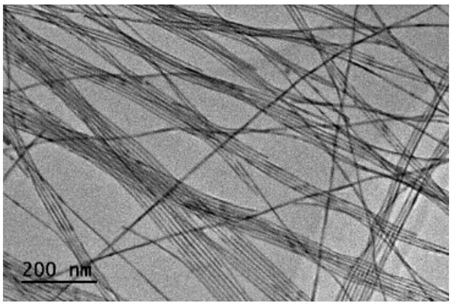

Figure S11. TEM image of the as-synthesized Pd nanowires.

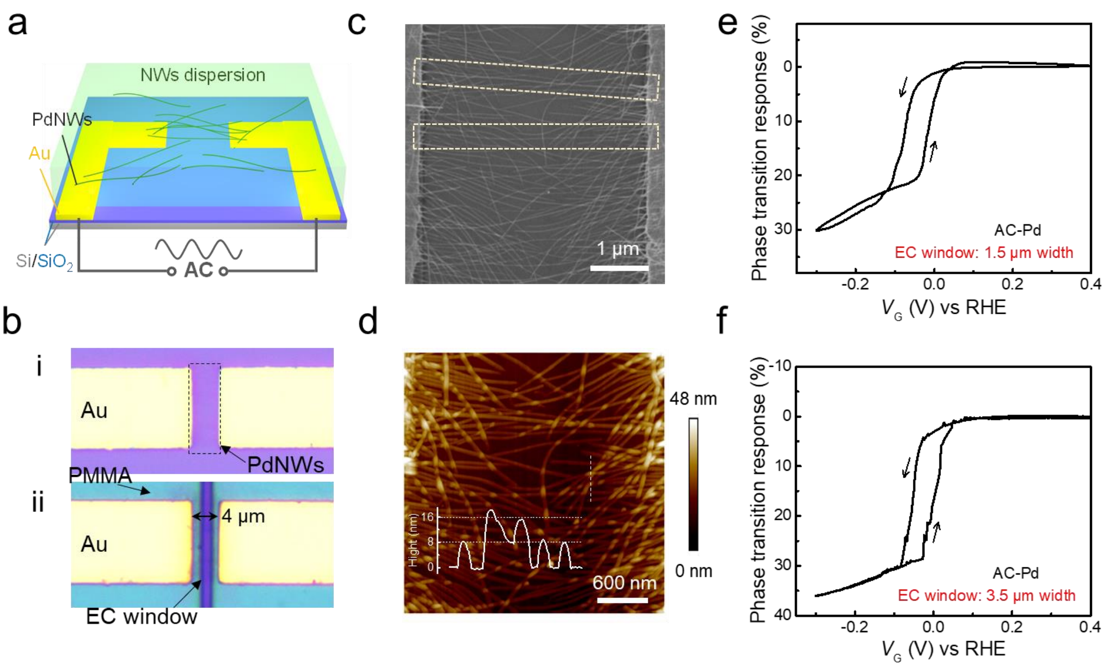

Figure S12. (a) The schematic experimental setup for deposition of Pd nanowires between two paralleled gold electrodes using alternating current (AC) dielectrophoresis. (b) Optical microscopic images of a device with Pd nanowires deposited between two paralleled gold electrodes (i) and exposed Pd nanowires in the electrochemical window (ii). The AC generator was operated at a frequency of 10 MHz and a peak-to-peak voltage ($V_{p\text{-}p}$) of 2.5 V for 10 min. (c, d) SEM (c) and AFM (d) images of the on-chip Pd nanowires in (b). The inset in (d) shows the AFM section height profiles of the on-chip Pd nanowires. (e, f) Phase transition responses of AC-Pd devices in 0.1 M $HClO_4$. Solid arrows in (e, f) indicate the potential sweeping direction.

Other factors that may affect phase transition response include surface contamination or substrate interaction[2]. For further revealing the substrate effect, we fabricated ultra-thin Pd devices with thickness of about 8~17 nm using alternating current dielectrophoresis and allow the individual nanowire to better contact with Si/SiO_2 substrate (Figure S12). Alternating current (AC) dielectrophoresis was initially developed for the separation of metallic from semiconducting single-

walled carbon nanotubes[3]. An AC electric field was used to induce dipole moment inside the nanotubes, which makes the nanotubes aligned in the direction of the electric field and attracted toward paired electrodes. While metals usually have small dielectric constants, AC electrophoresis is also suitable for the fabrication of devices of metal nanowires (Figure S12a). To fabricate Pd nanowire device by AC electrophoresis, 50 μL Pd nanowires suspension was first drop-casted onto the chip. The AC generator was then operated at a frequency of 10 MHz and a peak-to-peak voltage ($V_{p\text{-}p}$) of 2.5 V for 10 min. The large length (>1 μm, shown in Figure S11) of the as-synthesized Pd nanowires allows it to be directly attached between two electrodes (dashed rectangle in Figure S12c) with thickness of about 8~17 nm (Figure S12d). The phase transition responses of AC-Pd devices are 25.40~36.07 % (Figure S12e-f), which is lower than 33.34~50.00% obtained on Pd devices with thicknesses of 25±15 nm (Figure S9), indicating that the contact and strong interaction between the substrate and Pd can inhibit the volume expansion of the Pd during phase transition process[2].

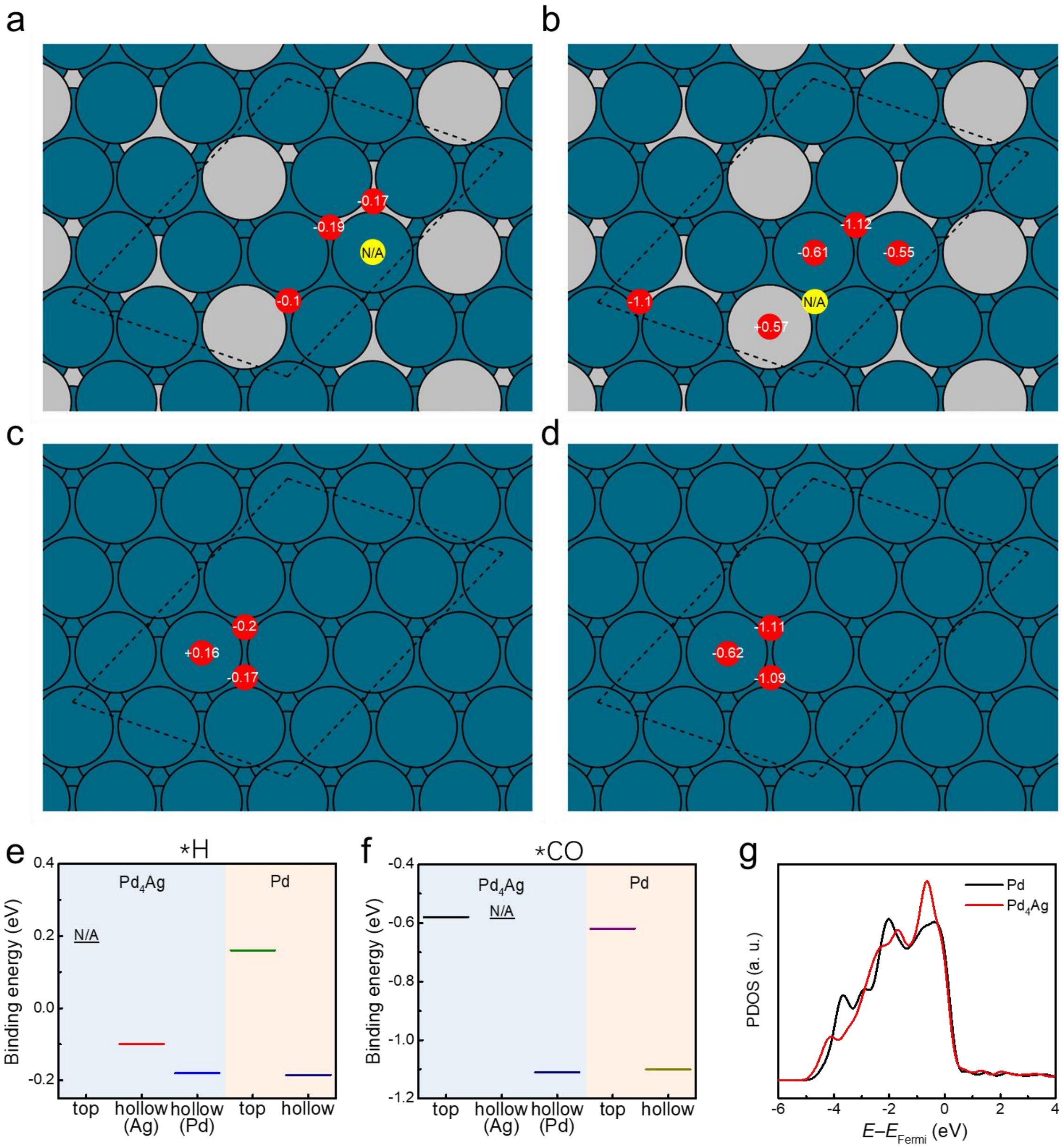

Figure S13. (a-d) Adsorption configurations of *H (a, c) and *CO (b, d) on Pd_4Ag (a, b) and pure Pd (c, d). The adsorption energies at different sites are listed in the red cycles. The unit of adsorption energy is “eV”. “N/A” in yellow cycles represent unstable adsorptions and their adsorption energies cannot be obtained after structure optimization. Pd atoms are blue and Ag atoms are grey. (e, f) Adsorption energies of *H and *CO on Pd_4Ag and Pd surfaces at different sites. (g) Partial density of states (PDOS) of *d* orbital of surface Pd atoms in Pd_4Ag and Pd.

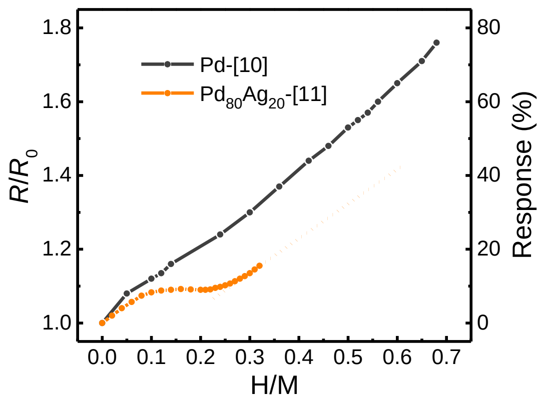

Figure S14. Resistivity and phase transition responses of pure Pd and $Pd_{80}Ag_{20}$ as a function of M/H ratio (x)[4, 5]. H/M ratio represents the ratio of hydrogen atoms to the combined total of Pd and Ag atoms.

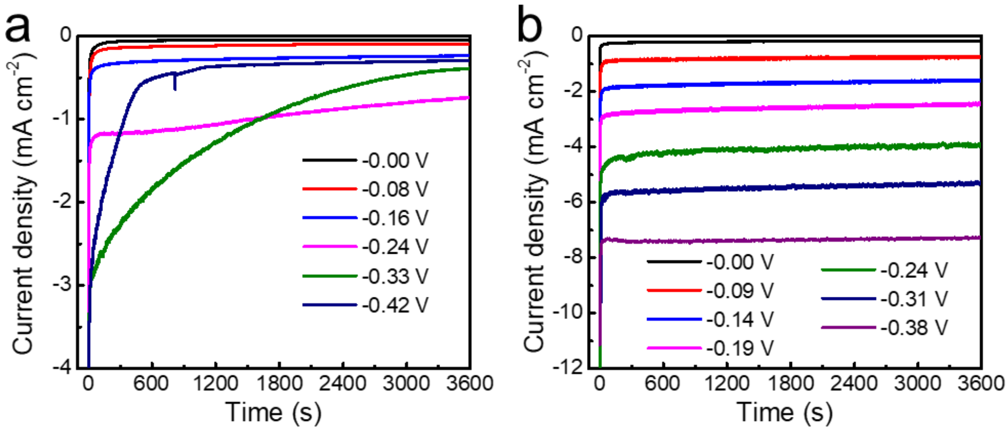

Figure S15. Chronoamperometric curves of Pd (a) and Pd_4Ag (b) in 0.1 M CO_2-saturated $KHCO_3$.

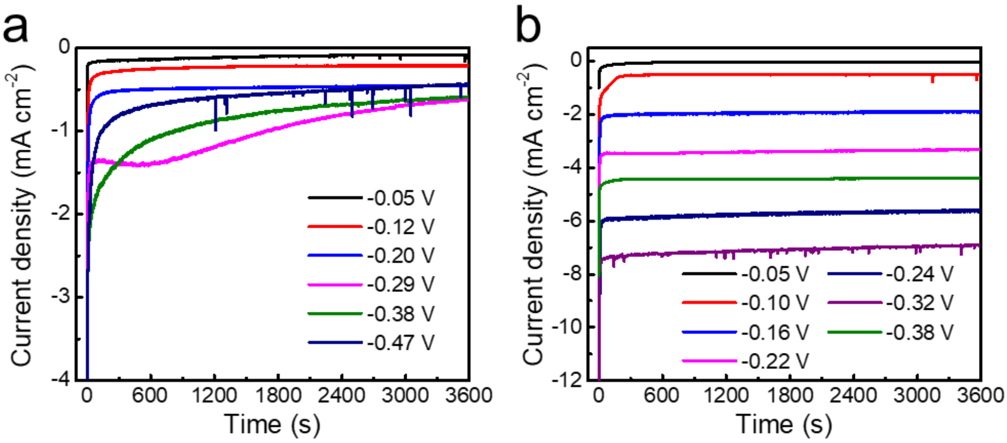

Figure S16. Chronoamperometric curves of Pd (a) and Pd_4Ag (b) in 0.1 M CO_2-saturated K_2HPO_4/KH_2PO_4.

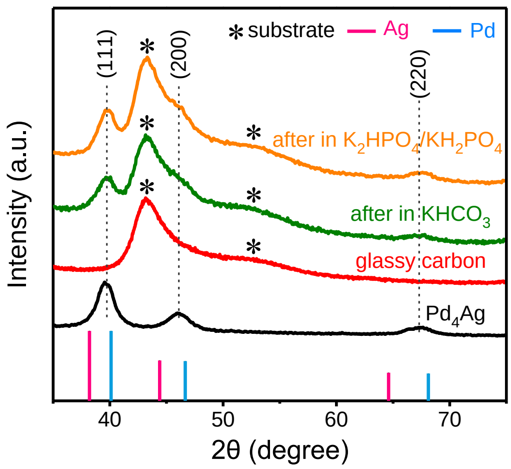

Figure S17. XRD patterns of the glassy carbon current collector (red curve), Pd_4Ag powders (black curve) and Pd_4Ag loaded on glassy carbon current collector after electrolysis at −0.38 V for 1 h in 0.1 M CO_2-saturated $KHCO_3$ (green curve) or K_2HPO_4/KH_2PO_4 (orange curve).

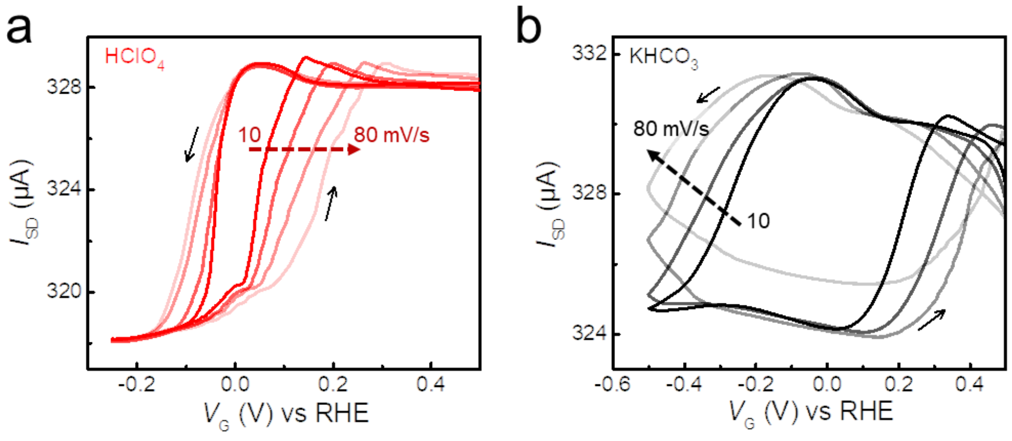

Figure S18. I_{SD}−V_G (ETS) curves of Pd in 0.1 M Ar-saturated $HClO_4$ (a) and $KHCO_3$ (b) with different scan rates at 10, 20, 40 and 80 mV/s. Film thickness is ~200 nm. Dashed arrows indicate the influence of scan rate (i.e., dynamic reaction time) on the hysteresis and degree of Pd phase transition. Solid arrows in all figures indicate the potential sweeping directions.

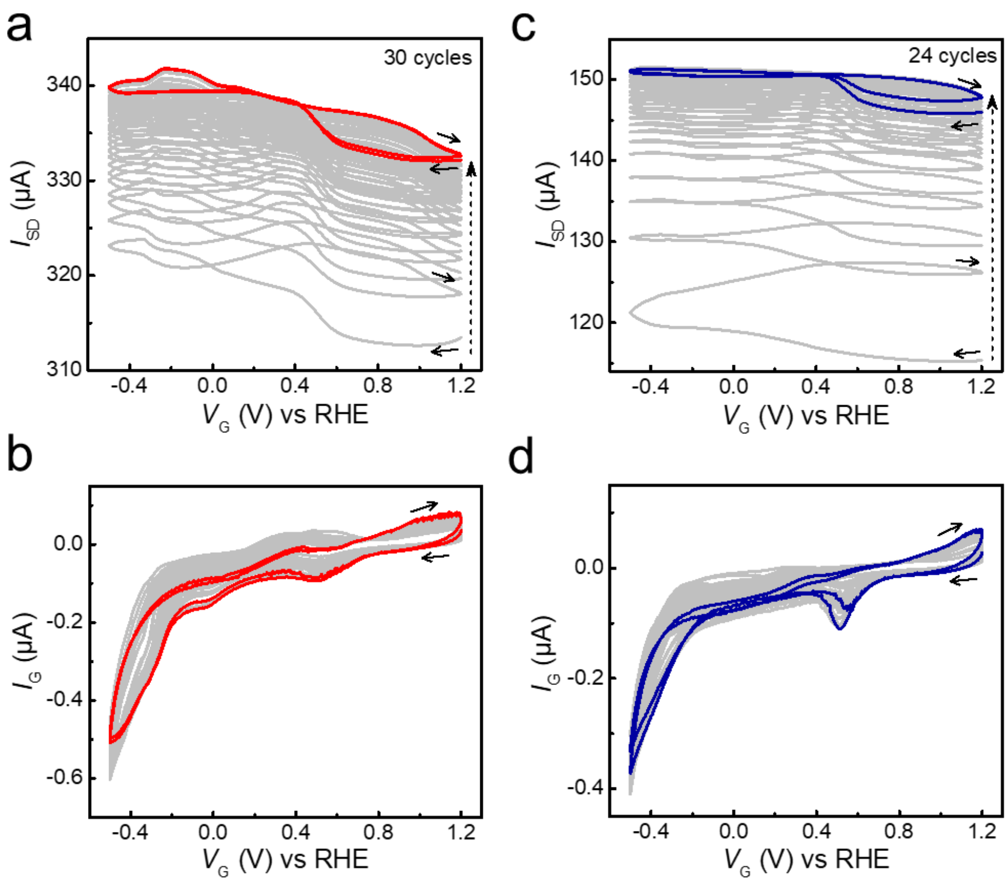

Figure S19. I_{SD}−V_G (ETS) and I_G−V_G (CV) curves (each sweep contains two potential cycles) of Pd (a, b) and Pd_4Ag (c, d) in 0.1 M CO_2-saturated $KHCO_3$. Film thickness is ~200 nm. Solid arrows in all figures indicate the potential sweeping direction.

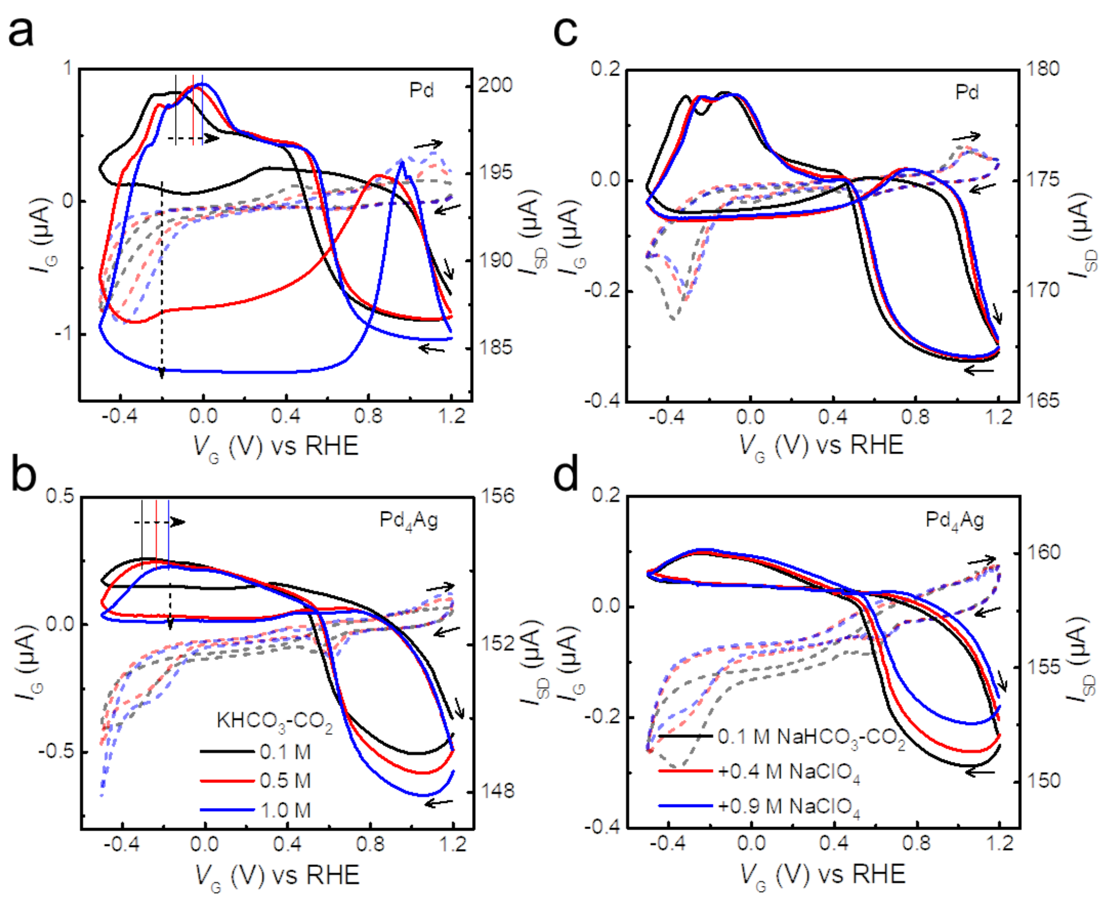

Figure S20. (a, b) I_{SD}−V_G (ETS) and I_G−V_G (CV) curves of Pd (a) and Pd_4Ag (b) in 0.1 M, 0.5 M and 1 M CO_2-saturated $KHCO_3$. (c, d) I_{SD}−V_G (ETS) and I_G−V_G (CV) curves of Pd (c) and Pd_4Ag (d) in 0.1 M CO_2-saturated $NaHCO_3$ and with addition of varying concentrations of Na^+. Film thickness is ~200 nm. In consideration of the low solubility of $KClO_4$ in water, $NaClO_4$ was used to study the cation effect. Solid arrows in all figures indicate the potential sweeping directions.

To further reveal the role of $KHCO_3$ in H sorption kinetics, ETS measurements were carried out in 0.1 M, 0.5 M and 1 M CO_2-saturated $KHCO_3$. The size of ETS hysteresis loop was found to increase in electrolytes with high concentrations (Figure S20a-b). Since these electrolytes are all near neutral with close pH values of 6.8, 7.3 and 7.6, the effects of ionic strength were considered here. Alkali cations are known to alter the structure of D.L. layer through the interfacial electric field and its

interaction with anions and intermediates[6-9]. However, by increasing the concentration of Na^+ in 0.1 M $NaHCO_3$ (Figure S20c-d), we found that cations cause little effect on phase transition. On the other hand, proton donor (HCO_3^-) was found to serve as the impacting factor to boost hydride formation in Pd (Figure S20a-b). The phase transition potential of pure Pd is at −8 mV_{RHE} in 1 M $KHCO_3$, which is highly consistent with previously reported value (close to 0 V_{RHE}) obtained by in situ XAS[10]. Correspondingly, the ETS provides relatively higher response for the measurements of H sorption processes, based on the working principle of phase transition induced electrical signals, which can be obtained with high precision.

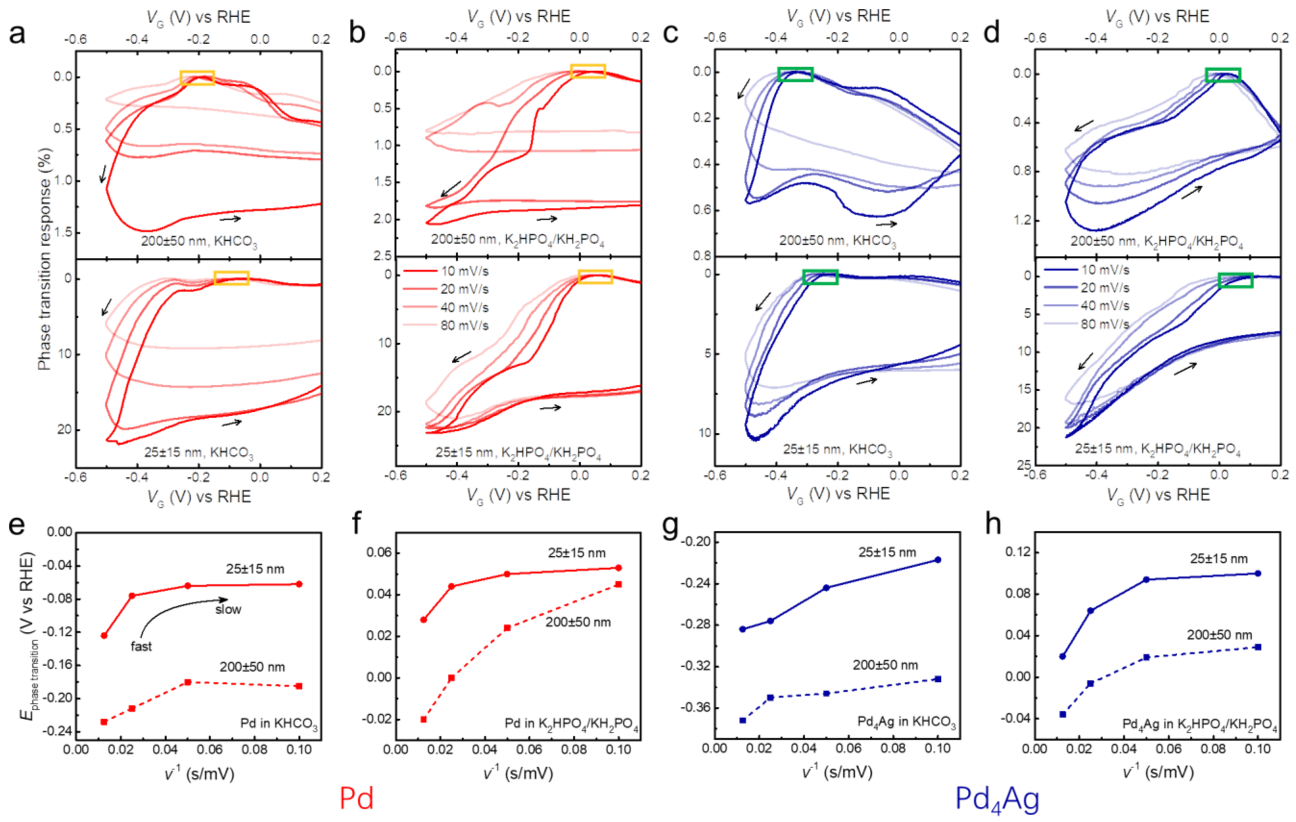

Figure S21. (a-d) Phase transition responses of Pd (a, b) and Pd_4Ag (c, d) in 0.1 M CO_2-saturated $KHCO_3$ (a, c) and K_2HPO_4/KH_2PO_4 (b, d) with different scan rates and film thicknesses. (e-h) Summarized dependence of onset potential for the phase transition of Pd (e, f) and Pd_4Ag (g, h) on the potential scan rate with different film thicknesses.

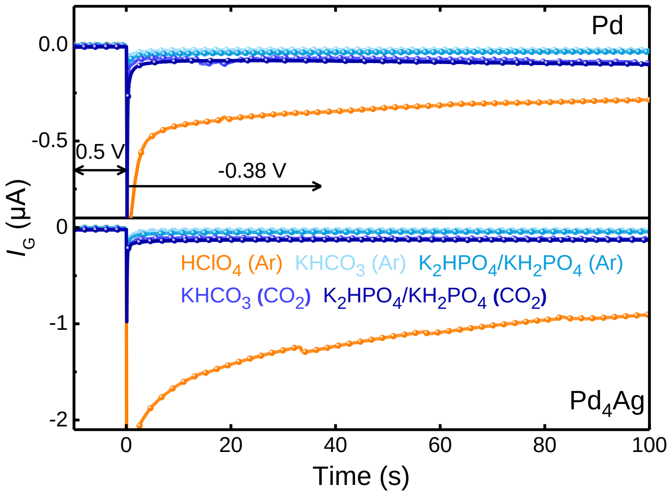

Figure S22. I_G–t curves of Pd and Pd_4Ag under potentiostatic conditions in different electrolytes with concentration of 0.1 M. The potential was first keeped at 0.5 V for 10 s and then shifted to −0.38 V for 100 s.

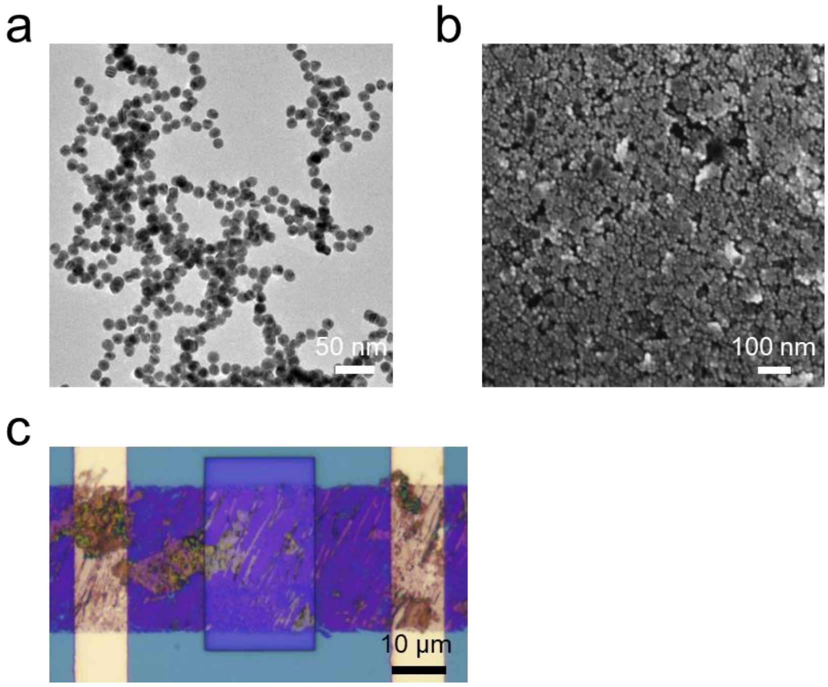

Figure S23. (a) TEM image of the as-synthesized Au nanoparticles. (b) SEM image of the on-chip film of Au nanoparticles. (c) Optical microscopic image of Au device.

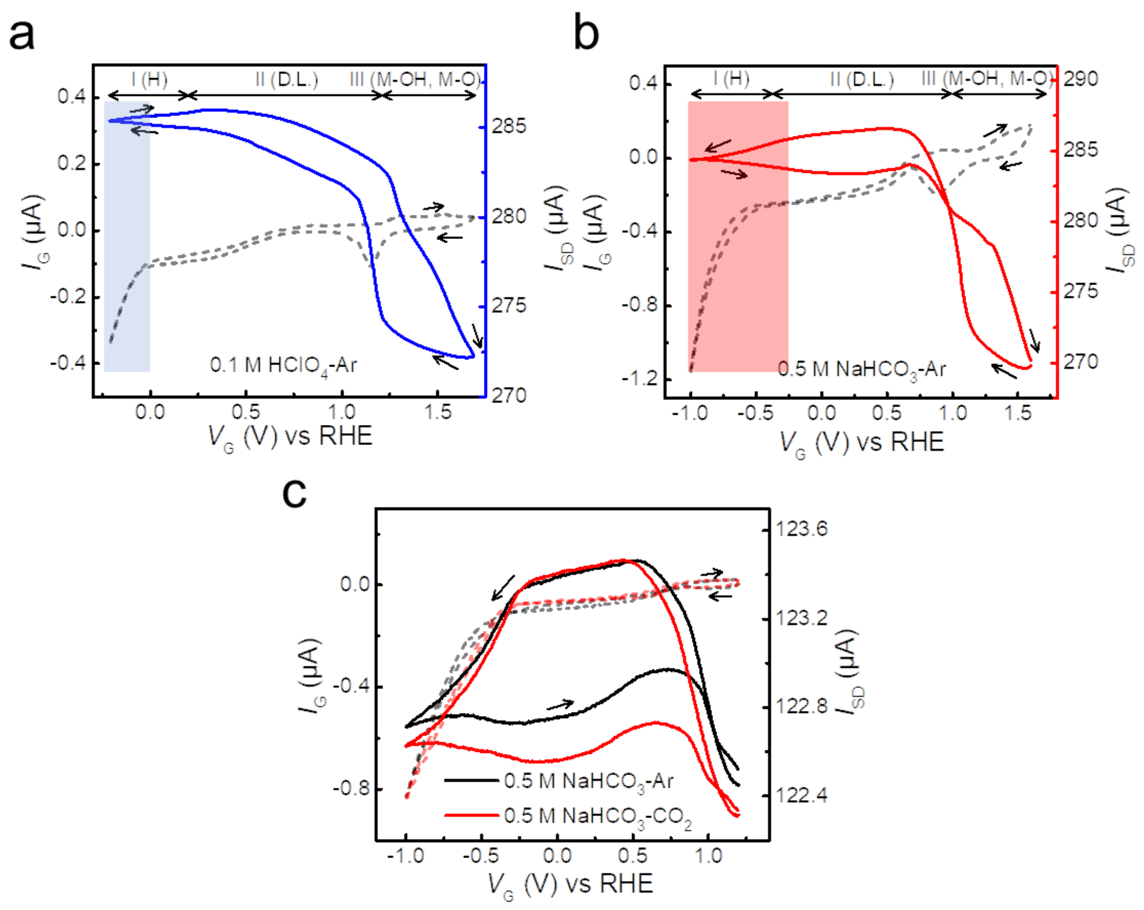

Figure S24. (a, b) I_{SD}–V_G (ETS) and I_G–V_G (CV) curves of Au in 0.1 M Ar-saturated $HClO_4$ (a) and 0.5 M Ar-saturated $NaHCO_3$ (b). (c) I_{SD}–V_G (ETS) and I_G–V_G (CV) curves of Au in 0.5 M Ar- and CO_2-saturated $NaHCO_3$. Solid arrows in all figures indicate the potential sweeping directions.

Hydrogen absorption (hydride formation) has been previously found to occur on Au electrodes[12-14], but has been rarely investigated probably due to the weak hydrogen production activity or high activation energy required for H_2 dissociation[15]. In our previous work, the surface adsorption features of Au in acid-base conditions have been systematically studied by ETS[11], and the measurements were all conducted at potentials higher than 0 V_{RHE} without obvious H phenomenon reflected on ETS. Here, we carried on ETS measurements of Au in $HClO_4$ and $NaHCO_3$ with potential extended to lower than 0 V_{RHE} (Figure S24).

As shown in Figure S24a&S24b, the ETS and CV curves obtained in 0.1 M $HClO_4$ and $NaHCO_3$ are divided into three regions: H adsorption/absorption/evolution region (region I), double-layer region (D.L., region II), and reversible adsorption of hydroxyl groups and surface oxide formation region (M−OH and M−O, region III). While the potential is scanned positively in 0.1 M $HClO_4$, the I_{SD} of region II shows obvious decrease due to anion (ClO_4^-) adsorption, and this phenomenon is not observed in 0.5 M $NaHCO_3$ due to the strong and stable OH^- adsorption at high pH.[11] While the potential is shifted to HER region, the ETS current in 0.5 M $NaHCO_3$ shows obvious decrease due to H absorption (red rectangle in Figure S24b). The H adsorption/absorption peaks of Au are usually hard to be identified on CV curves due to the weak H adsorption and slow H absorption rate on Au. Sustersic et al. loaded Au wire (with a length of 3 cm and a diameter of 1 mm) with H_{abs} by electrolysis at 0.1 V_{RHE} in 0.5 M H_2SO_4 for 6~360 min and then scanned the potential to 1.2 V_{RHE} to obtain an obvious H oxidation peak[14]. From our results, the ETS current shows slight increase with potential shifted to lower than 0.2 V_{RHE} in 0.1 M $HClO_4$ probably due to H adsorption, and ETS current does not decrease with the potential shifted to HER region (blue rectangle in Figure S24a), indicating the relatively weak H absorption in Au in acidic condition.

As shown in Figure S24c, the effect of CO_2RR on H absorption in Au was further studied in 0.5 M Ar- and CO_2-saturated $NaHCO_3$. The size of ETS hysteresis loop caused by H absorption is increased in CO_2-saturated $NaHCO_3$, indicating that the addition of CO_2 and H_2CO_3 increases the proton-donating capacity of the electrolyte and thus promotes H sorption kinetics. The inhibition effects on H sorption by CO_2RR is not dominant.

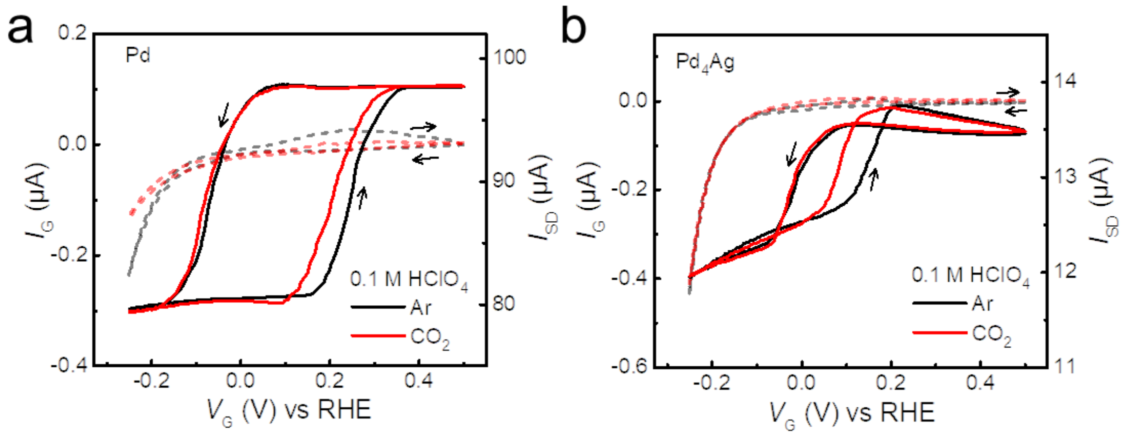

Figure S25. I_{SD}−V_G (ETS) and I_G−V_G (CV) curves of Pd (a) and Pd_4Ag (b) in 0.1 M Ar- and CO_2-saturated $HClO_4$. Film thickness is controlled to be less than 100 nm. While the hydrogen evolution on pure Pd is inhibited by CO_2RR, the phase transition process reflected on ETS is nearly unaffected due to the fast H sorption kinetics in acidic condition. Solid arrows in all figures indicate the potential sweeping direction.

Table S1. Adsorption energies of *H and *CO on Pd_4Ag and Pd surfaces at different sites. "N/A" represent unstable adsorptions and their adsorption energies cannot be obtained after structure optimization.

**Adsorbate**	***H (top)**	***H (hollow) (@Ag)**	***H (hollow) (@Pd)**
Pd	+0.16 eV	---	-0.17/-0.2 eV
Pd_4Ag	N/A	-0.1 eV	-0.17/-0.19 eV
**Adsorbate**	***CO(top)**	***CO (hollow) (@Ag)**	***CO (hollow) (@Pd)**
Pd	-0.62 eV	---	-1.09/-1.11 eV
Pd_4Ag	-0.61/-0.55 eV	N/A	-1.1/-1.12 eV

Table S2. Phase transition responses and H/M ratios of Pd and Pd_4Ag at −0.38 V_{RHE} for 100 s in 0.1 M CO_2-saturated $KHCO_3$ and K_2HPO_4/KH_2PO_4. The H/M ratios were obtained by normalizing the responses in Figure 4a with reference to the maximum phase transition responses (Pd: 45%, Pd_4Ag: 13%) obtained in 0.1 M $HClO_4$, and referring to the published quantitative relationship between resistivity and H/M ratios of pure Pd and $Pd_{80}Ag_{20}$ alloy (Figure S14). The errors present the standard error in two repeated ETS experiments.

	**ΔR_{MH_x} in $KHCO_3$**	**ΔR_{MH_x} in K_2HPO_4/KH_2PO_4**	**$\Delta(\Delta R_{MH_x})/\Delta R_{MH_x}$**	**H/M in $KHCO_3$**	**H/M in K_2HPO_4/KH_2PO_4**	**Δ(H/M) /(H/M)**
Pd	32.32±4.51%	34.39±3.89%	6.79±2.89%	0.48±0.01	0.51±0.02	6.25±4.17%
Pd_4Ag	10.71±0.15%	15.02±0.64%	40.05±3.88%	0.38±0.03	0.48±0.03	24.70±0.31%

References

1. Duncan, H., Lasia, A. Separation of hydrogen adsorption and absorption on Pd thin films. *Electrochim. Acta* ***53***, 6845−6850 (2008).

2. Lee, E., Lee, J. M., Koo, J. H., Lee, W., Lee, T. Hysteresis behavior of electrical resistance in Pd thin films during the process of absorption and desorption of hydrogen gas. *Int. J. Hydrogen Energy* ***35***, 6984−6991 (2010).

3. Krupke, R., Hennrich, F., v. Löhneysen, H., Kappes, M. M. Separation of metallic from semiconducting single-walled carbon nanotubes. *Science* ***301***, 344−347 (2003).

4. Barton, J. C., Lewis, F. A., Woodward, M. Hysteresis of the relationships between electrical resistance and the hydrogen content of palladium. *Trans. Faraday Soc.* ***59***, 1201–1207 (1963).

5. Tóth, J., Garaguly, J., Péter, L., Tompa, K. Resistivity changes during hydrogenation of $Pd_{80}Ag_{20}$ alloy in non-equilibrium circumstances. *J. Alloys Compd.* ***312***, 117–120 (2000).

6. Marcandalli, G., Goyal, A., Koper, M. T. M., Electrolyte effects on the Faradaic efficiency of CO_2 reduction to CO on a gold electrode. *ACS Catal.* ***11***, 4936−4945 (2021).

7. Strmcnik, D., Kodama, K., van der Vliet, D., Greeley, J., Stamenkovic, V. R., Markovic, N. M. The role of non-covalent interactions in electrocatalytic fuel-cell reactions on platinum. *Nat. Chem.* ***1***, 466−472 (2009).

8. Ringe, S., Clark, E. L., Resasco, J., Walton, A., Seger, B., Bell, A. T., Chan, K. Understanding cation effects in electrochemical CO_2 reduction. *Energy Environ. Sci.* ***12***, 3609−3610 (2019).

9. Resasco, J., Chen, L. D., Clark, E., Tsai, C., Hahn, C., Jaramillo, T. F., Chan, K., Bell, A. T., Promoter effects of alkali metal cations on the electrochemical reduction of carbon dioxide. *J. Am. Chem. Soc.* ***139***, 11277−11287 (2017).

10. Gao, D., Zhou, H., Cai, F., Wang, D., Hu, Y., Jiang, B., Cai, W.-B., Chen, X., Si, R., Yang, F., Miao, S., Wang, J., Wang, G., Bao, X. Switchable CO_2 electroreduction via engineering active phases of Pd nanoparticles. *Nano Res.* ***10***, 2181−2191 (2017).

11. Mu, Z., Yang, M., He, W., Pan, Y., Zhang, P., Li, X., Wu, X., Ding, M. On-chip electrical transport investigation of metal nanoparticles: characteristic acidic and alkaline adsorptions revealed on Pt and Au surface. *J Phys. Chem. Lett.* ***11***, 5798−5806 (2020).

12. Sustersic, M. G., Almeida, N. V., Von Mengershausen, A. E. Hydrogen oxidation on gold electrode in perchloric acid solution. *Int. J. Hydrogen Energy* ***35***, 6063−6068 (2010).

13. Martins, M. E., Podesta, J. J., Arvia, A. J. Chemical evidence of hydrogen sorption processes on potential cycled gold electrodes. *Electrochim. Acta* ***32***, 1013−1017 (1987).

14. Sustersic, M. G., Almeida, N. V., Von Mengershausen, A. E., Esquenoni, S. M. Hydrogen oxidation on gold electrode in sulphuric acid solution. *Int. J. Hydrogen Energy* ***37***, 14747-14752 (2012).

15. Greeley, J., Mavrikakis, M. Surface and subsurface hydrogen: adsorption properties on transition metals and near-surface alloys. *J. Phys. Chem. B 109*, 3460−3471 (2005).
